# Mast Cell Desensitization in Allergen Immunotherapy

**DOI:** 10.3389/falgy.2022.898494

**Published:** 2022-06-16

**Authors:** Celia López-Sanz, Rodrigo Jiménez-Saiz, Vanesa Esteban, María Isabel Delgado-Dolset, Carolina Perales-Chorda, Alma Villaseñor, Domingo Barber, María M. Escribese

**Affiliations:** ^1^Department of Immunology, Instituto de Investigación Sanitaria Hospital Universitario de La Princesa (IIS-Princesa), Madrid, Spain; ^2^Department of Immunology and Oncology, Centro Nacional de Biotecnología (CNB)-CSIC, Madrid, Spain; ^3^Faculty of Experimental Sciences, Universidad Francisco de Vitoria (UFV), Madrid, Spain; ^4^McMaster Immunology Research Centre (MIRC), Department of Medicine, McMaster University, Hamilton, ON, Canada; ^5^Department of Allergy and Immunology, Instituto de Investigación Sanitaria Fundación Jiménez Díaz (IIS-FJD), Universidad Autónoma de Madrid (UAM), Madrid, Spain; ^6^Faculty of Medicine and Biomedicine, Alfonso X El Sabio University, Madrid, Spain; ^7^Department of Basic Medical Sciences, Facultad de Medicina, Institute of Applied Molecular Medicine Nemesio Díez, Universidad San Pablo-CEU, CEU Universities, Urbanización Montepríncipe, Madrid, Spain; ^8^Centre for Metabolomics and Bioanalysis (CEMBIO), Department of Chemistry and Biochemistry, Facultad de Farmacia, Universidad San Pablo CEU, CEU Universities, Urbanización Montepríncipe, Madrid, Spain

**Keywords:** allergen immunotherapy, mast cell, desensitization, Treg, anaphylaxis, IgE

## Abstract

Allergen immunotherapy (AIT) is the only treatment with disease-transforming potential for allergic disorders. The immunological mechanisms associated with AIT can be divided along time in two phases: short-term, involving mast cell (MC) desensitization; and long-term, with a regulatory T cell (Treg) response with significant reduction of eosinophilia. This regulatory response is induced in about 70% of patients and lasts up to 3 years after AIT cessation. MC desensitization is characteristic of the initial phase of AIT and it is often related to its success. Yet, the molecular mechanisms involved in allergen-specific MC desensitization, or the connection between MC desensitization and the development of a Treg arm, are poorly understood. The major AIT challenges are its long duration, the development of allergic reactions during AIT, and the lack of efficacy in a considerable proportion of patients. Therefore, reaching a better understanding of the immunology of AIT will help to tackle these short-comings and, particularly, to predict responder-patients. In this regard, omics strategies are empowering the identification of predictive and follow-up biomarkers in AIT. Here, we review the immunological mechanisms underlying AIT with a focus on MC desensitization and AIT-induced adverse reactions. Also, we discuss the identification of novel biomarkers with predictive potential that could improve the rational use of AIT.

## Introduction

Allergic diseases are a heterogeneous group of immunological disorders characterized by a detrimental reaction to a given allergen. The onset of allergy occurs at the sensitization phase, which entails induction of a T helper (Th) 2 response and production of interleukin (IL)-4, IL-13 or IL-5, and immunoglobulin (Ig) E. Following sensitization, the effector phase is triggered by allergen re-exposure (1, 2). Effector allergic reactions are complex and often classified -according to the timing of the reaction- in acute and late phase (3, 4). The former is largely (but not exclusively) mediated by IgE (5–8) and its binding to the high-affinity IgE receptor (FcεRI), which is expressed on eosinophils (9, 10), monocytes (11), dendritic cells (12, 13) platelets (14), and specifically on basophils (15, 16) and mast cells (MCs) (17–19). IgE-FcεRI cross-linking leads to MC degranulation and the rapid release of vasoactive and pro-inflammatory mediators (e.g., histamine, tryptase or prostaglandins), which underlies clinical manifestations associated with acute allergic reactions, such as angioedema, hypotension or even anaphylaxis (1, 2, 5, 20, 21). Of these, anaphylaxis is defined as a life-threatening condition that compromises patient's airway, breathing, and/or circulation, and may occur without typical skin features or the presence of cardiovascular collapse (22).

The standard of care is allergen avoidance, when possible, together with the urgent treatment of an allergic reaction upon accidental allergen exposure (23). Allergen immunotherapy (AIT) is the most promising therapeutic approach as it is the only clinical intervention with disease-transforming capacity. AIT has been proven to confer long-term protection and to prevent disease progression and exacerbation. AIT operates in two phases: an early or escalation phase, headed by MC hypo-responsiveness on allergen provocation and an increase of Th2 cells and IgE; and a late or consolidation phase that takes 2–3 years of treatment and is dominated by regulatory T cells (Treg) (24–26). Nevertheless, effector cell activation and adverse side effects can happen at any time during AIT, compromising patients' safety and compliance. Thus, it is essential to discover reliable biomarkers to monitor immunological changes, to prevent side effects and to identify AIT-responder patients that can benefit from intervention.

Recent studies support that AIT efficacy relies on MC desensitization during the initial phase (24, 27, 28). However, the molecular mechanisms underlying AIT-induced MC hypo-responsiveness are controversial (29). Given that MC degranulation is a common driver of anaphylaxis (30), understanding MC desensitization is key, not only for preventing these life-threating reactions, but also for improving current intervention strategies, including AIT. Here, we review the immunological mechanisms underlying AIT with a focus on MC desensitization and AIT-induced adverse reactions. Also, we discuss the identification of novel biomarkers with predictive potential that could improve the rational use of AIT.

## The Immune Response Underlying AIT

AIT constitutes a pivotal pharmacological intervention aiming to control allergic diseases such as allergic rhinitis, allergic asthma, atopic dermatitis, insect venom hypersensitivity (31) or food allergy (29, 32). It consists of the administration of subsequent increasing doses of allergen until an adequate dose is reached, which induces immunological tolerance (31). The efficacy of AIT relies on changes in both innate and adaptive immune cells and is associated with a shift from a Th2 toward a Th1 and Treg phenotype. However, despite being in use for 110 years, the immunological mechanisms of AIT remain poorly understood (33).

**Figure 1:**
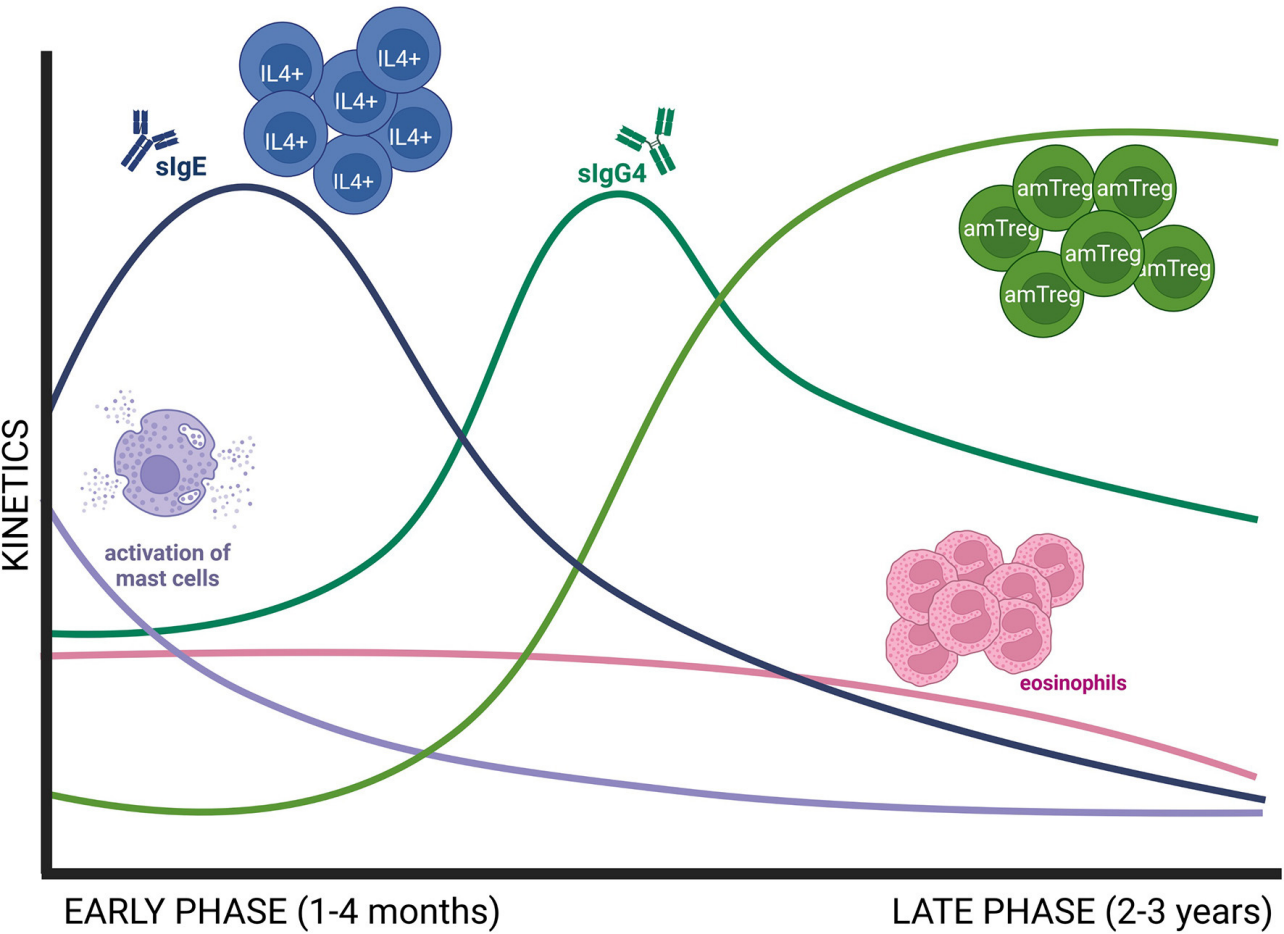
Schematic representation of early and late immune changes associated to AIT. The early phase of AIT (1–4 months) is dominated by a decrease in MC activation, known as desensitization, and an initial invigoration of Th2 immunity, with increasing levels of allergen sIgE and IL4^+^ cells. Then, sIgE decreases while sIgG4 increases at the end of the early phase. Consolidation of AIT needs 2–3 years of treatment and is defined by an increase in Treg responses and a decrease in Th2 immune responses as well as in peripheral eosinophilia. amTreg, active memory Treg; MC, mast cell.

A 3-year-follow-up study demonstrated that the immunological changes induced by sublingual AIT come about in two sequential phases (Figure 1). First, an early desensitization phase which takes place in the first 4 months. This stage is accompanied by an initial but short invigoration of Th2 immunity, with an increase in both allergen-specific Ig (sIg) E and IL4^+^ cells (24). In addition, AIT has been demonstrated to impair MC degranulation in this early stage (29, 32, 34). Finally, there is a later augmentation in sIgG/sIgG_4_ levels, which compete with sIgE and inhibit sIgE, thus preventing MC and basophil activation and their production of Th2-related cytokines (29).

Next comes the consolidation of the regulatory response, which needs at least 3 years of AIT (24). During this period there is a contraction of IL4^+^ cells, a downregulation of sIgE levels and a decrease in blood eosinophilia (24). In parallel, there is an increase in Treg responses, particularly activated memory Treg cells (24). Recent studies have pointed out that there is a regulatory network between MCs and Tregs. On the one hand, Treg suppresses MC activation by different mechanisms (i.e., IL-10 secretion, MC anergy via OX40L engagement) (29). On the other hand, in a food allergy model, desensitized MCs facilitated a Treg cell expansion in a IL-2-dependent manner (32).

Altogether, MCs appear to play key roles in both early and late phase AIT. As previous reports demonstrated strong benefits only few months after starting treatment (24), MC desensitization seems to be a key mechanism in keeping AIT efficacy.

## AIT-Induced Anaphylaxis

Local and systemic adverse reactions have been observed during AIT (22, 35). Of these, systemic reactions are described in ~1–4% of patients and can be mild to severe, anaphylaxis being the gravest (31). Over the years, diverse definitions of anaphylaxis have appeared in the literature with the purpose of improving its diagnosis and patients' management. Lately, the World Allergy Organization (WAO) depicted anaphylaxis as a potential life-threatening compromise of airways, breathing, and/or circulation, which may occur without typical skin symptoms or the presence of circulatory shock (22). These symptoms are usually developed within the first 30 min after AIT administration (31).

While the occurrence of adverse reactions in AIT is influenced by factors such as viral infections, fever, physical activity, non-steroidal anti-inflammatory drug use, hormonal changes, *etc*., the route of administration and allergen type are determinant. AIT with aeroallergens is usually administered subcutaneously and is less likely to induce anaphylactic reactions (36, 37). On the other hand, adverse allergic reactions including anaphylaxis are more common in AIT with food allergens (38). In terms of the route, subcutaneous AIT in peanut allergy is highly associated with anaphylaxis (39), but oral and sublingual AIT for peanut (and other food allergens) are clearly safer (40–44). Despite being safer, a recent systematic review and meta-analysis showed that the risk of anaphylaxis was significantly higher in peanut-allergic patients undergoing oral AIT than in those following allergen avoidance (45).

The classical pathway of anaphylaxis is IgE-mediated and involves MCs and basophils (5–8) and recently, omalizumab in combination with AIT has been proven to improve patients' outcome (46). However, IgE-independent mechanisms have also been described in murine models, and there is growing evidence of their importance in humans (5, 8, 47). These mechanisms involve IgG and platelet activating factor (PAF) release by neutrophils, basophils and macrophages (5), or complement activation. Non-immunological anaphylaxis can also occur through the direct stimulation of MC degranulation (48, 49) or by Mas-related G protein-coupled receptor X2 (MRGPRX2) expressed in MCs. In addition, the differential contribution of the endothelium to the pathophysiology of the anaphylaxis is being increasingly recognized, which adds another layer of complexity to this clinical manifestation (50).

Anaphylaxis severity is correlated to MC degranulation and the release of pro-inflammatory mediators (50–52). Intriguingly, anaphylactic mediators such as histamine are released during AIT without induction of anaphylaxis (53, 54), which insinuates that a certain level of MC activation may be required to achieve desensitization. MC desensitization is accomplished during the early-phase of AIT, and studies in murine models support that this process directs the immunological outcome of AIT (30). However, the molecular mechanisms of AIT involve several effector cell types (55, 56). Therefore, it is likely that different cellular and molecular microenvironments created between immune and non-immune cells modify the threshold of a detrimental inflammatory MC response during AIT.

## MC Desensitization Mechanisms

MCs are key effector cells in allergic disease for different reasons. They are immune sentinels located in mucosal and epithelial tissues, close to the vascular and lymphatic endothelium (57, 58). Because of this strategic distribution, MCs sense and respond promptly to allergens or pathogens (59, 60). Furthermore, MCs have a long lifespan as compared to its mobile analog, the basophil (61); retain surface IgE for months (62, 63); and can react to minute amounts of allergen (64). On activation, MCs degranulate rapidly because they are equipped with cytoplasmic granules (50–200 per MC) that contain preformed allergic mediators (60, 65).

In sensitized individuals, IgE-FcεRI complex clustering causes MC activation and degranulation. FcεRI activates several pathways through the immunoreceptor tyrosine-based activation motif in its cytoplasmic domain, e.g., Syk, PI3K/Akt, ERK and STAT6 (66, 67). These routes increase the intracellular calcium flux, which is crucial for exocytosis of preformed inflammatory mediators such as histamine or tryptase. Also, they activate the *de novo* synthesis of late-phase inflammatory cytokines (e.g., IL-6, TNF-α), prostaglandins, leukotrienes, and PAF, among others (29). The rapid release of these vasoactive and inflammatory mediators underlies clinical manifestations associated with acute allergic reactions (i.e., angioedema, hypotension or cardiovascular collapse and anaphylaxis) (5, 20, 21).

**Figure 2:**
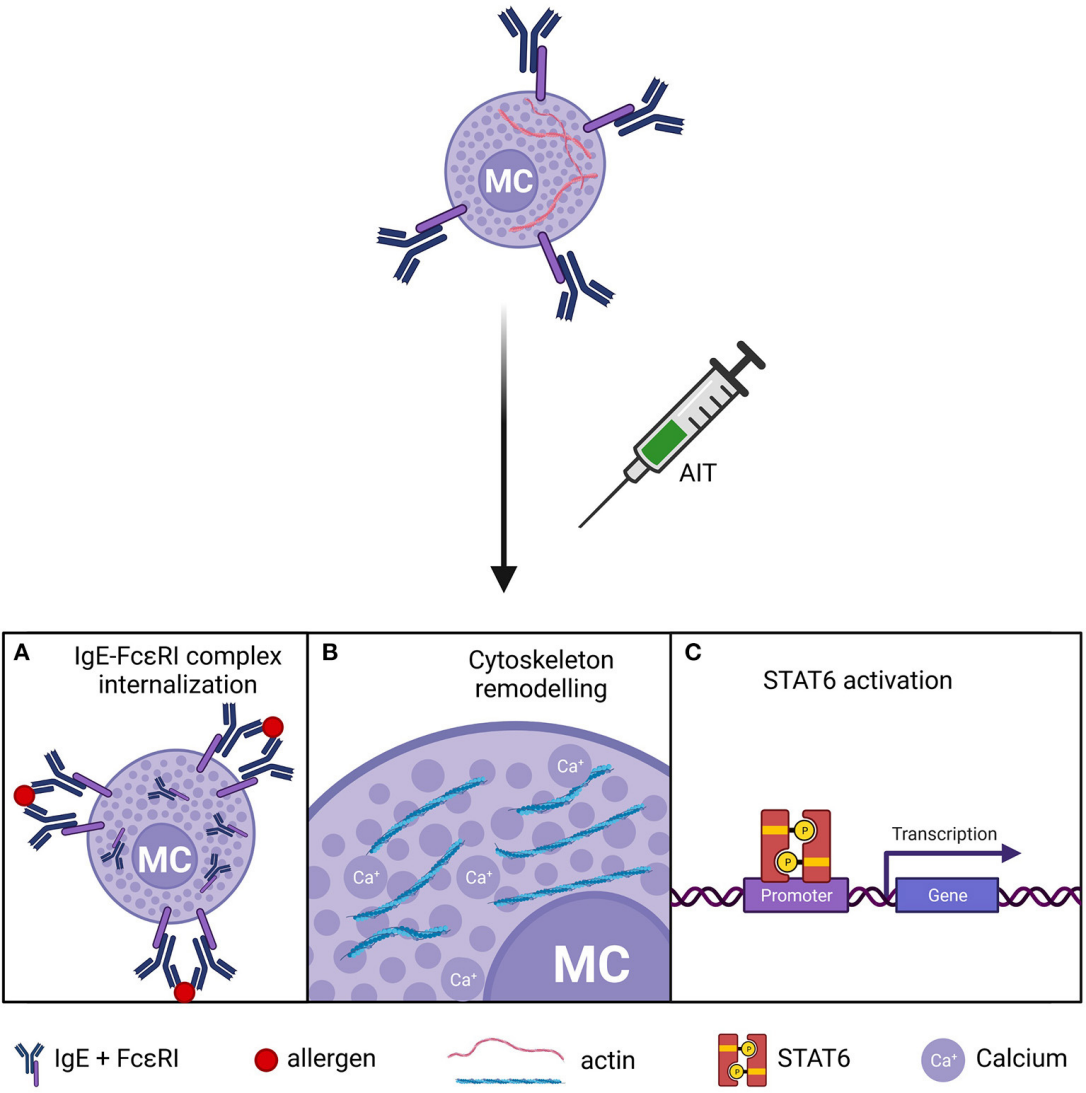
Putative mechanisms of MC desensitization in AIT. **(A)** Internalization of the IgE-FcεRI complex. **(B)** Actin-cytoskeleton remodeling and impaired calcium flux in MC after. **(C)** Dysregulation of STAT6 pathway. MCs, mast cells.

The IgE-MC pathway has long been a target for therapeutic intervention, and some drugs and biologicals have been developed to interfere with it (20, 29, 68). In this regard, AIT has been shown to dampen this axis (24, 26). Several *in vitro* and *in vivo* studies in mice have demonstrated that MC become hypo-responsive to allergen exposures after desensitization (34). MC desensitization appears to be allergen-specific (69, 70) and reversible (71, 72). Yet, the molecular mechanisms underlying AIT-induced MC desensitization remain controversial (29) (Figure 2).

Different *in vitro* studies support that increasing doses of allergen induce IgE-FcεRI complex internalization, rendering MCs unresponsive to allergen challenge (70, 73). In contrast, others report a partial IgE reduction in desensitized MCs (69, 72, 74, 75). In these studies, primary MCs of different origins were assayed, including murine and rat peritoneal MCs (70, 73), murine bone marrow-derived MCs (69, 74), and human lung MCs (72), which may explain this inconsistency. Moreover, MC sensitization was performed with different IgE clones such as SPE-7 (69, 74) and ε-26 (70). However, experiments with the same clone yielded divergent results (69, 70). Other experimental variables may have contributed to the discrepancy in IgE internalization. For instance, Oka et al. (70) used lower MC cellularity and a higher target dose of allergen than Sancho-Serra et al. (69), which may have facilitated IgE saturation and internalization in the former. Despite the variable results on IgE internalization, all these experimental approaches induced MC desensitization. In other words, MC desensitization is accomplished whether the loss of IgE is total or partial. Hence, mechanisms other than IgE-FcεRI internalization might be at play during MC desensitization. The recent research of Nagata et al. (75) report that the size of the IgE-FcεRI internalization complexes are responsible of MC desensitization success.

Another line of inquiry on the mechanisms of MC desensitization focused on the STAT6 pathway. Morales *et al*. reported that murine STAT6-deficient bone marrow-derived MCs failed to get desensitized (76), although they also showed that MC desensitization did not induce STAT6 phosphorylation (69). Additional experiments in peritoneal MCs from STAT6-null mice demonstrated that STAT6 was redundant for desensitization (70). STAT6 affects different aspects of MC biology, and its deficiency may cause unspecific effects depending on the maturity of MCs. For example, STAT6 is required for IL4-dependent responses (77), which increase FcεRI expression on MCs (18). Besides STAT6, recent studies are shedding light on the cytoskeletal dynamics that drive MC activation and desensitization (66, 78, 79). Gladys Ang et al. (74) showed that desensitized MCs had an atypical but stable redistribution of the actin cytoskeleton, which precluded calcium flux from intracellular stores and abrogated exocytosis of inflammatory granules.

There are some questions remaining on the mechanisms of MC desensitization. The notion that IgE-FcεRI complex internalization occurs to some degree during MC desensitization is well established, but how this process is regulated is largely unknown. In this regard, recent studies in murine MCs suggested that sIgG binding to FcγRIIB is required for IgE downregulation (80), and other MC inhibitory signaling pathways such as gp49B1/LILRB4 (81) may be also involved in MC desensitization. Nevertheless, *in vitro* experimentation supporting IgE-FcεRI complex internalization was conducted in the absence of IgG (69, 70, 74). Moreover, the role of STAT6 in MC desensitization is controversial (69, 76) and the kinetics of STAT6 phosphorylation during desensitization are not clearly defined.

From a broader perspective, the current mechanistic knowledge on MC desensitization can explain how it occurs at the cellular or local level. However, the fact that minute amounts of allergen can desensitize systemically in AIT, even by sublingual route (82–84), is certainly intriguing and points toward the participation of widespread, fast-acting systems. Further studies are necessary to understand how allergen desensitization operates at the level of an entire organism, as well as to identify biomarkers to monitor/predict successful MC desensitization in AIT.

## Identification of Biomarkers in AIT

A biomarker is any substance objectively measured that can be used as an indicator of biological/pathological processes, or pharmacologic responses to a therapeutic intervention. There is a lack of reliable biomarkers that can accurately reflect the clinical course or predict a positive response to AIT (85–87). Despite this dearth, there are some *in vivo* and *in vitro* biomarkers applied to monitor AIT safety and efficacy.

In the clinical practice, *in vivo* biomarkers such as skin prick test (SPT), intradermoreaction, nasal provocation and controlled exposure tests in chambers evaluate allergen-specific reactivity, which is expected to decrease after AIT (88). *In vitro* biomarkers are based on the cellular and humoral events that take place during AIT (89). Some widespread biomarkers are the determination of total IgE (tIgE) and sIgE. The latter is the gold-standard test for AIT patient selection. A high sIgE/tIgE ratio is predictive of positive responses to AIT (90, 91), although it has not been properly validated. AIT-induced desensitization correlates with a CD4^+^ T cell shift from Th2 towards a Th1 and Treg phenotype (Table 1). Also, sIgE increases during up-dosing but decreases during the maintenance phase, in parallel with a higher production of sIgG4, which suggests the development of a Treg response (24). AIT has also been shown to increase sIgA (113) and IL-10-producing innate-like lymphoid cells 2 (98).

**Table 1:** Potential biomarkers in AIT.

**Domains**	**Biomarkers**		**Effect after AIT**	**References**
***In vivo*** **biomarkers**	Allergen provocation test	SPT	**↓**	(92–94)
		ID		
		NPT		
		EEC		
**Antibodies**	IgE	sIgE	**↓**	(95)
		tIgE		
		sIgE/tIgE		
	IgG	Total	**↑**	(95)
		IgG_4_	**↑**	
		tIgG/IgG_4_	**↓**	
	IgA	sIgA	**↑**	(96)
*Serum inhibitory activity for IgE*	IgE FAB		**↓**	(95)
	ELIFAB			
**Cellular biomarkers**	Treg cells		**↑**	(97)
	Breg cells		**↑**	
	DC	DC2 (GATA3)	**↓**	
		DCreg (C1qA1)	**↑**	
	IL10^+^KLR^+^ILC2		**↑**	(98)
*Basophil activation*	CD63		**↓**	(99–101)
	CD203c		**↓**	
	Intracellular DAO		**↑**	
	Basophil histamine release		**↓**	
*MC activation*			**↓**	(102)
*Eosinophil activation*			**↓**	
**Cytokines and chemokines**	Th2	IL-4	**↓**	(103)
		IL-13		
		IL-9		
		IL-17		
		Eotaxin		
		TNF-α		
	Th1	IL-12	**↑**	(104)
		INFγ		
	Treg	IL-10	**↑**	(105)
		TGFβ		
**Omics science**	**Biomarkers**			**Reference**
*Genomics*	Identification of functional variants in atopy and asthma severity			(106)
*Epigenomics*	DNA methylation of FoxP3			(107)
	DNA methylation of Th cytokine genes			(108)
*Transcriptomics*	Th and Treg cytokine and chemokine transcripts			(109)
*Proteomics*	Molecular markers for four different monocyte-derived DC subclasses			(97)
*Metabolomics*	Hydroxyeicosatetraenoic acids (HETEs) during subcutaneous immunotherapy			(110)
	Effect of patient sensitization on the metabolic profile during sublingual immunotherapy			(26)
*Microbiomics*	Influence susceptibility to allergic diseases			(111)
**Others**	**Biomarkers**			**Reference**
*Immunophenotyping*	Th and Treg cells, IgG subclass and IgE expressing B cells, Breg			(112)

*SPT, skin prick test; ID, intradermal test; NPT, nasal provocation test; EEC, Environmental exposure chamber; DC, dendritic cells; IL, interleukin; TNF, tumor necrosis factor; INF, interferons; TGFβ, Transforming growth factor beta; forkhead box protein 3 (FoxP3); ILC, innate lymphoid cells*.

Other biomarkers for AIT efficacy are the assessment of the serum inhibitory activity of IgE, which can be measured by IgE-facilitated allergen binding (IgE-FAB) (85) or enzyme-linked immunosorbent-facilitated antigen binding assay (ELIFAB). IgE-FAB determines the binding of allergen-IgE complexes to B cells via the low-affinity IgE receptor (FcεRII or CD23). The decrease of IgE-FAB correlates with a positive clinical response to AIT (87). It has been reported that serum IgE-inhibitory activity persists for several years and is associated with long-term clinical efficacy (114). Moreover, *in vitro* assays, like the basophil activation test (BAT) (115), which measures lysosomal-associated proteins indicative of degranulation (e.g., CD63, CD203c) have been used to evaluate basophil suppression in AIT (85, 91, 116). Also, cytokines, chemokines and cellular markers have been applied for the study of AIT (Table 1).

During the last several years, omics have been applied in AIT research. Omics are techniques that use high-throughput approaches, each one correlating with a specific level of the system biology. Genomics, epigenomics, transcriptomics, proteomics, metabolomics (including lipidomics) and microbiomics could empower the identification of new diagnostic strategies for AIT (117) (Table 1). Genomics has been applied for the discovery of genetic variants that predispose to atopy (118) or affect asthma severity (119). Genetic variants that associate with good AIT outcomes could be used as biomarkers moving forward to stratify patients prior to treatment (28). Epigenomics studies have suggested that DNA methylation patterns, specifically in gene promoter regions associated with Forkhead box protein 3 (FoxP3), could inform of AIT progress (120, 121). Additionally, it has been proposed that the microbiota composition could influence AIT efficacy (111), which is another potential source of AIT biomarkers. Furthermore, transcriptomics and proteomics have been used to improve AIT patient selection through the characterization of allergen extracts, along with a profiling of IgE reactivity (113, 122, 123). Regarding metabolomics, a recent study demonstrated that the type of the patient's sensitization (mono- or poli-sensitized) is key in the clinical response to AIT (26). A different study focused on eicosanoid profiles showed that they increased at the beginning of AIT and then decreased after 1 to 3 years of AIT, decreasing at year 3 to levels below than baseline (110). Finally, techniques such as immunophenotyping using flow cytometry and mass spectrometry have allowed the parallel analysis of all cell subpopulations in a sample during AIT (124).

## Conclusion and Remarks

Despite the widespread use of AIT for more than 110 years, MC desensitization has just recently been identified as a key mechanism during the first 2 years of AIT. Yet, fundamental mechanisms associated with desensitization remain obscure. How a MC gets desensitized in an allergen-specific manner and how the desensitization pattern is transmitted throughout all barrier systems is certainly intriguing. MCs have a broad repertoire of signaling pathways. Due to the potential for inducing life-threatening reactions, research focus has always been on MC degranulation, perhaps overlooking their role as lipid-secreting mediators such as prostaglandins or leukotrienes. Moreover, MCs hypo-responsiveness, even without dampening Th2 responses, is effective not only in anaphylaxis prevention, but also for the control of allergic symptoms and reduction of medication usage (83, 125). This supports the key role of MC activation in allergic inflammation.

The sustained and disease-modifying effect of AIT is linked to the acquisition and epigenetic fixation of a regulatory phenotype. However, how the initial MC control predates the Treg response is unclear. Understanding this link is pivotal for the design of new AIT strategies aiming to avoid IgE-mediated reactivity. To date, no study with strict focus on Treg induction has proven to be effective. If effector cell desensitization governs AIT during the first 2 years of intervention, studies aiming to bypass effector cell activation should be planned for at least 3 years of intervention.

Different inflammatory routes have been described in anaphylaxis. AIT reduces IgE and likely impairs the classical pathway of anaphylaxis, but its effect on allergic reactions mediated by alternative pathways is debatable. Alternative routes could be activated during allergic sensitization (126), and might be relevant in pediatric anaphylaxis and AIT to foods. Should this be the case, AIT patient selection may benefit from novel biomarkers that classify patients according to the dominant inflammatory routes (127).

## Author Contributions

MME and DB designed the manuscript structure and participated in the writing and discussion. CLS, VE, and RJS participated in the design, writing, and discussion of the manuscript. CLS, RJS, CPC, AV, and MIDD collaborated in the writing, correction, and discussion of the manuscript. AV and CPC prepared the table. MME and MIDD participated in the design and preparations of the figures.

## Funding

This work was supported by ISCIII (PI18/01467 and PI19/00044), cofounded by FEDER Investing in your future for the thematic network and co-operative research centers and the thematic network RICORS “Red de Enfermedades Inflamatorias” (REI) RD21 0002 0008. This work was also supported by the Ministry of Science, Innovation and Universities in Spain (PCI2018-092930) co-funded by the European program ERA HDHL – Nutrition & the Epigenome, project Dietary Intervention in Food Allergy: Microbiome, Epigenetic and Metabolomic interactions DIFAMEM. MIDD is supported by FPI-CEU predoctoral fellowships. RJS's laboratory acknowledges the support received by the Severo Ochoa Program (AEI/SEV-2017-0712), FSE/FEDER through the Instituto de Salud Carlos III (ISCIII; CP20/00043), The Nutricia Research Foundation (NRF-2021-13), New Frontiers in Research Fund (NFRFE- 2019-00083), and SEAIC (BECA20A9). VE laboratory was supported by ISCIII (PI21/00158) cofounded by FEDER Investing in your future for the thematic network and co-operative research centres ARADyAL RD16/0006/0013 and SEAIC (19_A08).

## Conflict of Interest

The authors declare that the research was conducted in the absence of any commercial or financial relationships that could be construed as a potential conflict of interest.

## Publisher's Note

All claims expressed in this article are solely those of the authors and do not necessarily represent those of their affiliated organizations, or those of the publisher, the editors and the reviewers. Any product that may be evaluated in this article, or claim that may be made by its manufacturer, is not guaranteed or endorsed by the publisher.

## Abbreviations

AIT: allergen immunotherapy
BAT: basophil activation test
ELIFAB: enzyme-linked immunosorbent-facilitated antigen binding assay
FcεRI: high-affinity IgE receptor
FcεRII: low-affinity IgE receptor
FoxP3: Forkhead box protein 3
IgE-FAB: IgE-facilitated allergen
Ig: immunoglobulin
IL: interleukin
ITAM: immunoreceptor tyrosine-based activation motif
MC: mast cell
MoAb: monoclonal antibodies
MRGPRX2: Mas-related G protein-coupled receptor X2
PAF: platelet activating factor
sIg: specific immunoglobulin
SPT: skin prick test
STAT6: signal transducer and activator of transcription 6
Th: T helper cells
tIgE: total IgE
TNF-α: tumor necrosis factor alfa
Treg: regulatory T cells.
